# A Longitudinal Study Investigating Whether Chronic Rhinosinusitis Influences the Subsequent Risk of Developing Dementia

**DOI:** 10.3390/jpm14111081

**Published:** 2024-10-30

**Authors:** Dae-Soon Son, Jae-In Kim, Dong-Kyu Kim

**Affiliations:** 1 Department of Data Science and Data Science Convergence Research Center, Hallym University, Chuncheon 24252, Republic of Korea; biostat@hallym.ac.kr; 2 Department of Physiology, Neurology, Hallym University, Chuncheon 24252, Republic of Korea; m23527@hallym.ac.kr; 3 Department of Otorhinolaryngology-Head and Neck Surgery, Chuncheon Sacred Heart Hospital, Hallym University College of Medicine, Chuncheon 24252, Republic of Korea; 4 Institute of New Frontier Research, Division of Big Data and Artificial Intelligence, Chuncheon Sacred Heart Hospital, Hallym University College of Medicine, Chuncheon 24252, Republic of Korea

**Keywords:** rhinosinusitis, dementia, incidence, risk, cohort study

## Abstract

**Background/Objectives:** Numerous studies have explored the association between chronic rhinosinusitis (CRS) and cognitive decline. However, whether CRS is an independent risk factor for the development of dementia remains unclear. Thus, this retrospective cohort study sought to examine the potential association between CRS and increased incidence and risk of dementia by utilizing a representative population-based cohort dataset. **Methods:** In this study, we identified 2126 patients with CRS aged >55 years and matched them with 8504 controls to assess the incidence and risk of dementia. **Results:** We found that the incidence of all-cause dementia in CRS patients was 0.125 per 1000 person-years. The risk of developing all-cause dementia events (adjusted hazard ratio [HR] = 1.0, 95% confidence interval = 0.8–1.3) also did not differ significantly between the control group and the CRS group, irrespective of the CRS phenotype. Subgroup analysis also showed no increased adjusted HR for developing Alzheimer’s disease (0.9, 0.7–1.2), Parkinson’s disease (0.9, 0.5–1.4), and other types of dementia (1.0, 0.7–1.4) in the CRS group compared to the control group. **Conclusions:** Therefore, the present study demonstrated that patients over 55 years of age with CRS did not exhibit an increased incidence or risk of dementia compared to individuals without CRS.

## 1. Introduction

Chronic rhinosinusitis (CRS) is a heterogeneous, prolonged inflammatory condition that impacts the mucosal tissues of the sinonasal cavities. It is characterized by persistent inflammation in the sinonasal pathways and manifests as two or more nasal and sinus symptoms that last for over 12 weeks consecutively [1]. It presents with a spectrum of symptoms ranging from rhinologic manifestations such as persistent nasal discharge, nasal obstruction, and facial pressure or pain to systemic effects including fatigue, mood disturbances, impaired sleep, diminished social interaction, and subjective reports of neurocognitive decline [2]. The degree of CRS condition was evaluated using nasal endoscopy and computed tomography, which have a weak correlation with the severity of the patient’s symptoms. Additionally, despite undergoing comprehensive medical and surgical interventions, some patients with CRS continue to experience recurrent and refractory symptoms, making the management of this condition particularly difficult [3]. Patients with CRS, particularly those with accompanying systemic conditions, tend to experience notably greater levels of pain, reduced energy consumption, and increased difficulty in performing daily activities. Therefore, CRS patients usually have a significantly diminished quality of life (QOL), decreased occupational performance, and considerable direct and indirect healthcare and societal costs [3,4,5].

Dementia is a neurodegenerative disorder recognized as a significant contributor to cognitive decline, leading to reduced independent functioning. Although the pathophysiological mechanisms underlying neurodegenerative diseases remain poorly understood, numerous studies have emphasized the role of different inflammatory processes in the brain as contributing elements to their development [6,7,8]. Several studies demonstrated that chronic inflammatory conditions were associated with cognitive impairment, as evidenced by findings in diseases such as sarcoidosis, sickle cell anemia, obesity, and various other disorders [9,10,11]. In addition, a population-based cohort study revealed a higher likelihood of cognitive impairment in individuals exhibiting persistently elevated or progressively increasing levels of interleukin-6 [12]. Other studies have demonstrated a link between persistent inflammation and a heightened risk of cognitive deterioration [13,14]. Consistent with these findings, several CRS studies have reported a potential connection between the onset of cognitive impairment and CRS [15,16,17]. In addition, olfactory dysfunction, a prevalent symptom of CRS, strongly has been linked to an increased risk of developing dementia, as reported in recent studies [18,19]. However, another study revealed that CRS was not associated with neurodegenerative dementia [20]. Therefore, the relationship between CRS and the development of dementia remains unclear and requires further investigation.

In this study, we aimed to investigate the relationship between CRS and the risk of developing dementia. To do so, we utilized a nationally representative cohort of 1,025,340 individuals from the 2002–2013 National Sample Cohort of the Korea National Health Insurance Service. This large-scale population-based dataset, which offers detailed medical service usage histories for over a million Koreans, enabled us to examine the association between CRS and dementia risk while accounting for both clinical and demographic factors.

## 2. Materials and Methods

### 2.1. Cohort Dataset

National health insurance has provided universal coverage to the entire South Korean population since 1989. Accordingly, all South Korean citizens are enrolled in the Korea National Health Insurance Service, with nearly all healthcare data systematically recorded in centralized databases. This system facilitates the regulation of medical expenditures among beneficiaries, healthcare providers, and the government. This nationwide population-based longitudinal cohort database encompasses nearly all medical data, including diagnostic codes, treatments, prescriptions, and personal demographic information. In this dataset, diagnostic classifications followed the International Classification of Diseases, 10th Revision, Clinical Modification (ICD-10-CM), ensuring consistent and accurate categorization of diseases. Each individual within the cohort was assigned a unique birth identifier, preventing the possibility of claim duplication or omission, thereby maintaining the dataset’s integrity. This rigorously constructed dataset offers an accurate representation of the entire adult population in South Korea during a specified period, significantly reducing the potential selection bias. It supports detailed monitoring of healthcare service utilization and outcomes, enabling a thorough analysis of healthcare trends and patterns. Thus, it provides essential insights into the prevalence of diseases, treatment efficacy, and healthcare-seeking behaviors. The representativeness of this dataset makes it well-suited for a broad spectrum of epidemiological research and health policy evaluations, providing significant insights for public health strategies and intervention planning in South Korea. Furthermore, the longitudinal nature of the dataset enables long-term studies, allowing researchers to track changes in health status and healthcare utilization over time. This feature is especially valuable for identifying trends and forecasting future healthcare needs. Due to its extensive scope and high level of detail, this dataset is an essential resource for researchers, policymakers, and healthcare professionals aiming to enhance the quality of care and improve the efficiency of the healthcare system in South Korea.

### 2.2. Ethical Considerations and Data Accessibility

This study utilized a nationally representative cohort dataset from the National Health Claims Database of the Korean National Health Insurance Service. This covers a significant portion of the population, including more than one million adults, representing roughly 2.2% of the total South Korean population, with excellent stability and minimal loss to follow-up. The study received approval from the Institutional Review Board (IRB) of Hallym University Chuncheon Sacred Hospital. The IRB number of the present study is 2021-08-006. Since the dataset comprised de-identified secondary data, the IRB waived the requirement for written informed consent. The full dataset cannot be made publicly available in compliance with privacy regulations established by the Korea National Health Insurance Corporation. However, all relevant data supporting the findings of this study are included in the analysis. Additional data may be requested for further examination, with access subject to institutional approval and adherence to data protection protocols.

### 2.3. Study Design

**Figure 1:**
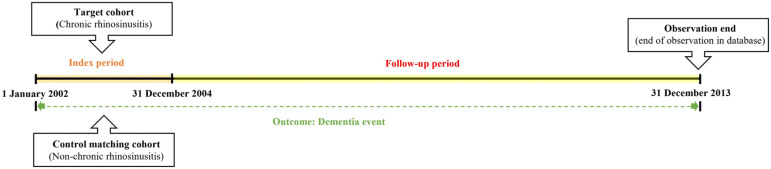
A detailed description of the study design and methodology.

**Figure 2:**
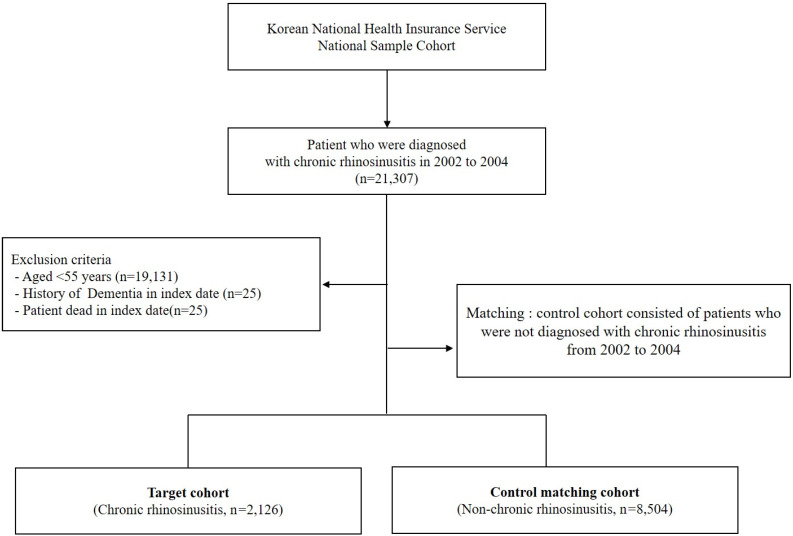
Schematic representation of the study enrollment process.

We first established the index period from 2002 to 2004 and identified patients diagnosed with CRS within this timeframe. CRS patients were identified using the diagnostic codes J32 (CRS without nasal polyps, CRSsNP) and J33 (CRS with nasal polyps, CRSwNP). Participants were included in the study if they had received these diagnostic codes on at least two separate occasions during the index period or had been hospitalized with a CRS diagnosis. For the purposes of this study, chronic rhinosinusitis was defined as having symptoms persisting for at least 12 weeks. To enhance the validity of the findings, patients under the age of 55, those who died during the index period, and individuals with a prior dementia diagnosis were excluded from the analysis. Control participants were selected using propensity score matching, with four non-CRS individuals matched to each CRS patient. The primary endpoint of the study was the onset of dementia by the end of the follow-up period. Dementia was classified as Alzheimer’s disease (F00, G30), Parkinson’s disease (G20), and other dementias (F02, F03). Additionally, all-cause mortality was considered an endpoint for the follow-up period. Patients who did not experience an event by the end of the follow-up were censored. Figure 1 illustrates the overall study design, while Figure 2 outlines the selection process for both groups (CRS vs. control).

A key aspect of the study design was controlling for potential confounding factors. To achieve this, we considered age, sex, residence, household income, and comorbidities as independent variables that could act as confounders, and we adjusted for these factors in both the CRS and control cohorts. Specifically, patient data including age, sex, residence, household income, and comorbidities were extracted from the database. The study population was categorized into two age groups (55–69 years and >69 years), and household income was classified as low (≤30.0% of the national median), middle (30.1–69.9% of the national median), and high (≥70.0% of the national median). Residential areas were divided into Seoul (the largest metropolitan area in Korea), other metropolitan cities, small cities, and rural areas. Comorbidities such as hypertension (I10, essential hypertension), diabetes (E10–E14), and chronic kidney disease (N18) were identified using diagnostic codes recorded between 2003 and 2005, prior to dementia onset.

### 2.4. Statistical Analysis

In this study, we estimated the dementia incidence rate per 1000 person-years from the time of patient registration to each endpoint. We evaluated the dementia incidence rates in patients with and without CRS and conducted statistical analyses to compare the two groups, calculating the risk ratio of CRS in relation to dementia incidence. The association between CRS and comparison group characteristics was investigated with a chi-squared test. Confounding factors were adjusted using multivariate-adjusted logistic regression, Cox proportional hazards, and propensity scoring. Specifically, incidence was calculated to measure the frequency of a disease or specific event over a defined period, expressed per 1000 person-years. We counted person-years as follows: in the case of death, the period from the first CRS diagnosis to the date of death; in the case of an event, the period from the first CRS diagnosis to the date of the first occurrence of that event; and in the case of no event, the period from the first CRS diagnosis to the end of the study. Next, to assess whether patients with CRS were at an elevated risk of developing certain diseases, we conducted Cox proportional hazards regression analyses to estimate the hazard ratio (HR) and corresponding 95% confidence interval (CI), adjusting for other confounding variables. All statistical analyses were conducted using the Windows version of R software (version 4.0.0; R Foundation for Statistical Computing, Vienna, Austria). Two-sided *p*-values were calculated, and a *p*-value of less than 0.05 was considered to indicate statistical significance.

## 3. Results

### 3.1. Cohort Sampling for the Control and CRS Groups

A total of 8504 participants without CRS and 2126 patients with CRS aged >55 years were included in this study over a 12-year follow-up period. The CRS cohort comprised 879 men (41%) and 1247 women (59%). The demographic and clinical characteristics of the study population in each group are presented in Table 1.

All independent variables showed similar distributions between the control and CRS groups. This indicated that each variable was appropriately matched between the two matched cohort groups.

### 3.2. Incidence Analysis of Dementia Between the Control and CRS Groups

To determine the incidence rates, we evaluated 4207 person-years in the control group and 1492 person-years in the CRS group (Table 2). The incidence of all-cause dementia in the CRS group was 0.125 per 1000 person-years, while it was 0.122 per 1000 person-years in the control group.

### 3.3. Risk Analysis of Subsequent Development of Dementia Between the Control and CRS Groups

Univariate and multivariate Cox regression models were used to analyze the HR for the occurrence of incident dementia events in patients with CRS over the 12-year follow-up period (Table 2). We observed no significant difference in the risk ratio of incident all-cause dementia events between the two groups during the 12-year follow-up period (adjusted HR = 1.0, 95% CI = 0.8–1.3). Moreover, when we analyzed the risk of dementia onset over time by year from the time of CRS diagnosis, there was no period in which the risk of dementia onset increased significantly in a statistically significant manner (Table 3).

### 3.4. Subgroup Analysis of Subsequent Development of Dementia

Since females have a higher lifetime risk of developing dementia than males [21,22], we performed a risk analysis of dementia events according to sex (Table 4).

We found no significant sex differences in the development of all-cause dementia events between the two groups. Subsequently, we conducted further analysis of the risk ratio for dementia events based on the CRS phenotype (Table 5).

CRS is typically categorized into two phenotypes based on nasal endoscopic findings: CRSsNP and CRSwNP. We found that the risk rate of all-cause dementia was not significantly different between the control and CRS groups, regardless of the CRS phenotype. Additionally, we performed a subgroup analysis according to the dementia subtype (Table 6).

We found that the incidences of Alzheimer’s and Parkinson’s disease was 0.142 per 1000 person-years, and other dementia types were 0.114, 0.142, and 0.132 per 1000 person-years, respectively, in the CRS group. The incidences of Alzheimer’s disease, Parkinson’s disease, and other types of dementia were 0.117, 0.135, and 0.123 per 1000 person-years, respectively, in the control group. Moreover, we detected that our subgroup analysis indicated no significant difference in the risk rate of developing Alzheimer’s disease, Parkinson’s disease, and other dementia-type events between the two groups (adjusted HR = 0.9 [0.7–1.2]; adjusted HR = 0.9 [0.5–1.4]; adjusted HR = 1.0 [0.7–1.4], respectively).

## 4. Discussion

To date, CRS is usually classified into CRSsNP and CRSwNP based primarily on the detection of nasal polyposis under nasal endoscopic views. Although this classification has the advantage of being simple and intuitive, it does not fully reflect the differences in the underlying pathophysiological immune response. [23,24,25,26]. CRSsNP is a variable chronic inflammatory condition. The mainstay treatment includes intranasal corticosteroids and nasal irrigation with antibiotics reserved for the treatment of acute exacerbations. Additionally, some clinicians employ long-term macrolide therapy because of its combined antimicrobial and anti-inflammatory properties [27]. Oral corticosteroids are generally not recommended for the treatment of CRSsNPs unless type 2 predominant inflammation is strongly suspected. ESS is often performed in patients who do not respond to medical management, and endoscopic sinus surgery is often pursued [27]. Thus, CRS is one of the chronic inflammation diseases, and some studies showed the association between chronic inflammation and cognitive decline [12,13,14]. Dementia is a neurodegenerative disorder widely recognized as a leading contributor to cognitive decline, resulting in a significant reduction in independent functioning. Previous research has identified cognitive impairment in patients with CRS, but these studies are limited by their cross-sectional design rather than a longitudinal approach [15,16,17]. Moreover, they are constrained by the lack of a control group in their analysis. To address these limitations, we structured the current study as a retrospective cohort study and examined the CRS group using a matched control group for comparison. Therefore, in this study, we used a 12-year longitudinal population-based dataset and selected participants matched for sociodemographic factors. This allowed us to conduct a retrospective cohort analysis to evaluate the risk of dementia among patients with CRS, categorized by CRS phenotype. Our results demonstrated no significant correlation between CRS and an increased incidence of all-cause dementia, irrespective of the CRS phenotype. In addition, subtype analyses revealed no relationship between CRS and specific forms of dementia, including Alzheimer’s disease, Parkinson’s disease, and other dementia types.

Dementia is the most economically burdensome adult neurological disorder and ranks as the second leading cause of mortality from neurological diseases, following cerebrovascular diseases [28]. It is characterized by gradual deterioration in cognitive and behavioral functions, leading to significant impairment in performing daily tasks. The clinical presentation of dementia ranges from mild cognitive impairment to severe memory loss. It is associated with a broad spectrum of neurological conditions, such as Alzheimer’s and Parkinson’s diseases. Among dementia, Alzheimer’s dementia is an age-associated neurodegenerative disorder marked by several neuropathological features, including the presence of extracellular amyloid-β plaques, intracellular neurofibrillary tangles, inflammation, synaptic dysfunction, and neuronal loss, all contributing to cognitive decline. Parkinson’s disease is also an age-associated neurodegenerative disorder characterized by the progressive deterioration of specific regions of the brain. It is most commonly recognized by symptoms such as bradykinesia, tremors, balance impairments, and other motor deficits. Although most cases have idiopathic origins, a subset is linked to hereditary factors. Generally, it is well-known that modifiable risk factors of dementia include cognitive inactivity and social isolation. In contrast, non-modifiable risk factors associated with dementia include age, genetic polymorphisms, sex, family history, and ethnicity [29].

Brain inflammation can have profound acute and long-term consequences. Recent research has shown that neurodegeneration can arise from inflammation caused by the activation of the immune cells in brain tissues, which release a variety of pro-inflammatory mediators that harm neuronal health [30,31]. These studies indicate that in Alzheimer’s disease and Parkinson’s disease, inflammation is not merely a consequence of neurodegeneration but also plays a key role in driving the process [30,31]. Additionally, cognitive changes become increasingly prevalent with advancing age. Aging is the primary risk factor for cognitive decline, as it is inherently associated with increased physiological inflammation [8]. Other studies demonstrated that neuroinflammation can have both acute and long-term consequences. In addition to causing immediate localized damage through neurotoxic inflammatory mediators, prolonged neuroinflammation is associated with an increased risk of dementia [8,32,33]. This suggests that chronic inflammation may increase the risk of cognitive decline, thereby contributing to the development of dementia.

CRS is a heterogeneous, long-standing inflammatory disorder affecting the sinonasal passages, and it is defined by the presence of two or more symptoms indicative of sinonasal inflammation. Historically, CRSwNP has been associated with type 2 inflammation, whereas CRSsNP has been associated with type 1 inflammation. Studies analyzing inflammatory mediators in patients with CRS have revealed notable differences in cytokine expression, initially across geographical regions and, more recently, within traditional CRS phenotypes. In American and European cohorts, CRSwNP was predominantly characterized by a type 2 immune response. However, this pattern is not reflected in Asian populations, where CRSwNP, particularly in China and Korea, tends to be associated with neutrophilic inflammation [34,35,36]. Despite the appropriate performance of endoscopic sinus surgery, some patients with CRSwNP continue to experience persistent symptoms and disease recurrence and may require revision surgeries [37]. Additionally, several previous studies suggested that CRS is associated with an increased incidence of depression and anxiety [38,39,40]. Moreover, CRS patients are known to experience sleep disturbance, and it often leads to the development of sleep apnea [41]. Sleep apnea is also closely linked to a substantially higher risk of developing dementia, especially in relation to Alzheimer’s and Parkinson’s diseases [42]. Due to these overlapping pathophysiological processes, we proposed that CRS patients might be more likely to develop dementia, and we carried out this study to explore this possible connection. However, our longitudinal analysis demonstrated that CRS in patients aged >55 years was not associated with an increased risk of developing dementia, irrespective of the CRS phenotype, sex, or dementia subtype. We propose that the discrepancy between our findings and previous studies may stem from the fact that cognitive decline in CRS patients is more likely the result of a complex interplay of multiple factors, rather than being directly caused by CRS alone.

This study possesses several notable strengths. First, although numerous studies have explored the association between CRS and comorbidities, many have been limited by their cross-sectional designs or relatively short follow-up periods. To overcome this limitation, we used a large national population-based database with an extended follow-up period of 12 years. Second, our findings have important clinical implications. The extended follow-up period enabled a thorough examination of the potential link between CRS diagnosis and the long-term development of dementia. In particular, performing concurrent extra-sinonasal evaluations during the management of patients with CRS could facilitate the early detection and timely treatment of dementia. Third, this study aimed to assess differences in dementia risk among patients with CRS based on specific phenotypes. CRS is a highly heterogeneous condition. However, clinical phenotypes alone do not fully capture its pathophysiological diversity. However, increasing evidence from several studies indicates that CRSsNP is primarily associated with a type 1 inflammatory response, whereas CRSwNP is associated with type 2-driven inflammation and elevated eosinophil infiltration. Therefore, understanding the variations in the risk of dementia between these phenotypes is crucial for advancing personalized medicine in this domain.

However, this study has some clear limitations. First, we were unable to obtain data on the severity of CRS, such as nasal symptom scores and radiological imaging findings. This prevented us from further analyzing the changes in outcomes by severity. To mitigate this limitation, we applied the operational definition of CRS described in the Methods section. Second, due to the study design, some non-CRS patients may have developed CRS during the follow-up period between 2005 and 2013. Third, we did not have access to certain personal health information, including body mass index, lipid profile, or behavioral risk factors such as smoking and alcohol consumption. These may also be potential confounders. Fourth, medications commonly used in the treatment of CRS, such as antihistamines, steroids, and leukotriene antagonists, may have played a role in managing chronic inflammation. However, we were unable to match the use of these medications between the two groups. To address this limitation, we applied propensity score matching to select a comparison group (non-CRS participants) with sociodemographic similarity to the CRS group. Fifth, investigating the relationship between CRS and the increased risk of dementia is critical, as factors like family history, genetic predisposition, and radiological findings on magnetic resonance imaging may affect the likelihood of developing Alzheimer’s disease or Parkinson’s disease. Therefore, our results may be influenced by confounding factors. Lastly, we did not examine the underlying pathophysiological mechanisms that connect CRS and dementia. Future research should consider a wider range of factors, diagnostic criteria, and objective assessments of CRS severity to further elucidate the link between CRS and dementia.

In conclusion, this study evaluated whether CRS in patients aged 55 years and older affects the subsequent risk of developing all-cause dementia, Alzheimer’s disease, or Parkinson’s disease. Our findings indicate that CRS in this age group was not associated with an increased risk of any of these neurodegenerative conditions. Moreover, in the subgroup analysis, the risk of all-cause dementia did not differ according to sex in patients with CRS. Furthermore, neither CRS phenotype, including CRSsNP or CRSwNP, was significantly associated with the risk of developing all-cause dementia.

## Figures and Tables

**Table 1:** Characteristics of the study participants.

Variables	Control (*n* = 8504)	CRS (*n* = 2126)	*p*-Value
Sex			0.9
Male	3516 (41%)	879 (41%)	
Female	4988 (59%)	1247 (59%)	
Ages			0.9
55–69	5852 (69%)	1463 (69%)	
>69	2652 (31%)	663 (31%)	
Residence			0.9
Seoul	2244 (26%)	561 (26%)	
Second area	2356 (28%)	589 (28%)	
Third area	3904 (46%)	976 (46%)	
Household income			0.9
Low (0–30%)	1676 (20%)	419 (20%)	
Middle (30–70%)	1888 (22%)	472 (22%)	
High (70–100%)	4940 (58%)	1235 (58%)	
Comorbidity			0.9
No	4248 (50%)	1062 (50%)	
Yes	4248 (50%)	1062 (50%)	

CRS: chronic rhinosinusitis; three residential regions (Seoul, the largest metropolitan area in South Korea; other metropolitan cities; and small cities and rural areas); household income (low [≤30.0% of the national median], middle [30.1–69.9% of the national median], and high [≥70.0% of the national median]).

**Table 2:** Incidence and risk of all-cause dementia (Alzheimer’s disease, Parkinson’s disease, and other types of dementia) in the control and CRS groups.

Variables	N	Case	Person-Year	Incidence	Unadjusted HR (95% CI)	Adjusted HR (95% CI)
All-cause dementia						
Control	8504	512	4207	0.122	1.00 (ref)	1.00 (ref)
CRS	2126	186	1492	0.125	1.1 (0.9–1.3)	1.0 (0.8–1.3)
Alzheimer’s disease						
Control	8504	252	2148	0.117	1.00 (ref)	1.00 (ref)
CRS	2126	88	770	0.114	1.0 (1.8–1.3)	0.9 (0.7–1.2)
Parkinson’s disease						
Control	8504	78	577	0.135	1.00 (ref)	1.00 (ref)
CRS	2126	37	260	0.142	1.1 (0.7–1.6)	0.9 (0.5–1.4)
Other types of dementia						
Control	8504	182	1483	0.123	1.00 (ref)	1.00 (ref)
CRS	2126	61	462	0.132	1.2 (0.9–1.6)	1.0 (0.7–1.4)

CRS: chronic rhinosinusitis; HR: hazard ratio; CI: confidence interval.

**Table 3:** Hazard ratios for incident all-cause dementia events in patients with CRS by time since CRS diagnosis.

Time (Year)	Number of All-Cause Dementia		Adjusted HR (95% CI)	*p*-Value
	Comparison	CRS		
1	NA	NA	NA	NA
2	NA	1	NA	NA
3	13	3	0.3 (0.01–9.5)	0.5055
4	50	17	0.7 (0.4–1.4)	0.3597
5	72	33	0.6 (0.4–0.9)	0.0285
6	106	49	0.9 (0.6–1.2)	0.3779
7	165	66	0.9 (0.6–1.2)	0.3477
8	218	88	0.8 (0.6–1.1)	0.1956
9	301	114	1.0 (0.8–1.3)	0.7432
10	364	133	1.1 (0.9–1.3)	0.4698
11	424	157	1.0 (0.8–1.2)	0.9063
12	512	186	1.0 (0.8–1.1)	0.6059

CRS: chronic rhinosinusitis; HR: hazard ratio; CI: confidence interval.

**Table 4:** Hazard ratio of all-cause dementia event by sex between comparison and CRS group.

Sex	Male		Female	
	Comparison	CRS	Comparison	CRS
Unadjusted HR (95% CI)	1.00 (ref)	1.1 (0.8–1.5)	1.00 (ref)	1.1 (0.8–1.3)
Adjusted HR (95% CI)	1.00 (ref)	1.0 (0.7–1.3)	1.00 (ref)	1.0 (0.7–1.2)

CRS: chronic rhinosinusitis; HR: hazard ratio; CI: confidence interval.

**Table 5:** The incidence and the risk of dementia events according to the CRS subtype.

Variables	N	Case	Person-Year	Incidence	Unadjusted HR (95% CI)	Adjusted HR (95% CI)
All-cause dementia						
Comparison	8504	512	4207	0.122	1.00 (ref)	1.00 (ref)
CRSsNP	1864	162	1289	0.126	1.13 (1.0–1.4)	1.0 (0.81–1.2)
CRSwNP	262	24	203	0.118	0.9 (0.6–1.3)	0.8 (0.5–1.2)

CRS, chronic rhinosinusitis; CRSsNP, chronic rhinosinusitis without nasal polyps; CRSwNP, chronic rhinosinusitis with nasal polyps; HR, hazard ratio; CI, confidence interval.

**Table 6:** The incidence and the risk of dementia subtypes, including Alzheimer’s disease, Parkinson’s disease, and other types of dementia between control and CRS groups.

Variables	N	Case	Person-Year	Incidence	Unadjusted HR (95% CI)	Adjusted HR (95% CI)
Alzheimer’s disease						
Control	8504	252	2148	0.117	1.00 (ref)	1.00 (ref)
CRS	2126	88	770	0.114	1.0 (1.8–1.3)	0.9 (0.7–1.2)
Parkinson’s disease						
Control	8504	78	577	0.135	1.00 (ref)	1.00 (ref)
CRS	2126	37	260	0.142	1.1 (0.7–1.6)	0.9 (0.5–1.4)
Other types of dementia						
Control	8504	182	1483	0.123	1.00 (ref)	1.00 (ref)
CRS	2126	61	462	0.132	1.2 (0.9–1.6)	1.0 (0.7–1.4)

CRS: chronic rhinosinusitis; HR: hazard ratio; CI: confidence interval.

## Data Availability

The datasets generated and/or analyzed in the current study are not publicly available owing to the policy of the Korea National Health Insurance Service but are available from the corresponding author upon reasonable request.
